# Study of Thyroid Function in Children With Beta-Thalassemia Major

**DOI:** 10.7759/cureus.86772

**Published:** 2025-06-25

**Authors:** Mrunalini Kulkarni, Rahul V Kawade, Vinay S

**Affiliations:** 1 Department of Paediatrics, Bharati Vidyapeeth (Deemed to be University) Medical College and Hospital, Sangli, IND; 2 Paediatric Critical Care, Bangalore Baptist Hospital, Bangaluru, IND

**Keywords:** beta thalassemia, euthyroid, hypothyroidism, serum ferritin, thyroid function

## Abstract

Beta-thalassemia major is an autosomal-recessive hereditary hemolytic anemia. The homozygous state results in severe anemia, which needs frequent blood transfusions. It eventually leads to iron overload causing multiple complications like hypogonadism, hypothyroidism, hypoparathyroidism, diabetes mellitus, liver fibrosis, and cardiac dysfunction. Iron overload leads to increased morbidity and mortality in transfusion-dependent thalassemia children. Thyroid dysfunction in thalassemia is primary hypothyroidism.

Methodology

This was an observational analytical study. The children enrolled were between two and 18 years old, admitted to the thalassemia day-care unit over a period of six months. The study protocol was approved by the Institutional Ethical Committee.

Results

Forty beta-thalassemia major patients were included in the present study, of which 18 (45%) patients were between six and 10 years old, 15 (37.5%) patients were between 11 and 18 years old, and seven (17.5%) patients were between two and five years old. Twenty-four (60%) patients were boys and 16 (40%) were girls. Serum ferritin and thyroid function were measured in these 40 study patients, in which 27 (67.5%) patients were euthyroid, eight (20%) patients had subclinical hypothyroidism, and five (12.5%) patients had overt hypothyroidism. In this study, patients with serum ferritin >2000 ng/ml, 50% were euthyroid and 50% were hypothyroid (19.23% had overt hypothyroidism and 30.77% had subclinical hypothyroidism). So there was a positive correlation between serum ferritin and hypothyroidism. As serum ferritin increased, thyroid-stimulating hormone (TSH) also showed an increasing trend and was statistically significant (p<0.05).

Conclusion

Detection of hypothyroidism is important as effective replacement therapy is available. Therefore, thyroid function should be evaluated periodically, particularly in iron overload cases. Early recognition and treatment will help to improve quality of life in thalassemia patients.

## Introduction

The terminology thalassemia is obtained from Greek word *thalassa* meaning sea and *haima* meaning blood. It was originally found in countries surrounding the Mediterranean Sea. It denotes a group of hematological disorders with absent or reduced synthesis of one of the two types of polypeptide chains (alpha or beta), which form the normal adult hemoglobin (Hb A). It ultimately leads to reduced hemoglobin in red cells and severe anemia [1].

It is calculated that, in the world, there are about 180-200 million people with carriers of B-thalassemia gene. They comprise about 1.5% of the total world population. Forty million of them are present in Southeast Asia. Out of them, 20 million carriers are present in India alone. Every year, 80,000 to 100,000 children are born with thalassemia worldwide. In India, 8000-10,000 children with thalassemia are born every year. The prevalence of beta-thalassemia carrier state is 1%-3% in South India and 15%-20% in North India [2].

Thalassemia is inherited in autosomal-recessive manner. It's hallmark is defective synthesis of hemoglobin, ineffective red cell production and their increased breakdown, leading to multiple complications like cardiac failure and death in early life [3].

The severe form of thalassemia results in severe anemia. It needs repeated packed cell transfusions to maintain hemoglobin levels in the normal range. However, these repeated transfusions may result in iron excess and its toxicity. Iron overload mainly manifests as multiple endocrine problems like hypothyroidism, diabetes mellitus, and hypogonadism. Excess iron in the liver leads to liver fibrosis and cirrhosis. Cardiac iron excess will cause cardiomyopathy, arrhythmia, and myocardial dysfunction [4]. Thyroid dysfunction in thalassemia is primary hypothyroidism. Subclinical hypothyroidism takes at least 10 years to manifest. It is seen in about 13%-60% of patients [4].

Iron overload leads to increased morbidity and mortality in transfusion-dependent thalassemia children. Iron chelation treatment has significantly improved the survival and quality of life of these children. At the same time, longer survival is associated with multiple endocrine and non-endocrine complications [5].

However, with optimal chelation, children have disorders related to growth or delayed puberty. These are due to dysfunction of hypothalamopituitary axis [6].

This study was conducted to evaluate thyroid function in children with beta-thalassemia as early detection of hypothyroidism and treatment with thyroxine will probably lead to a better outcome.

## Materials and methods

Study design

This observational analytical study was conducted in the Thalassemia Day Care Unit of a tertiary care medical college hospital.

Study population

All children who were brought for packed red blood cell transfusion in a period of six months were included in the study. Informed consent or assent was taken.

Sample size

Forty children fulfilling the inclusion criteria were included in the study over a period of six months.

Study subjects

All children fulfilling the exclusion and inclusion criteria presented to the thalassemia unit. Inclusion criteria were all patients aged two to 18 years of age admitted to the Thalassemia Day Care Unit for packed red blood cell transfusion. Children younger than two years and older than 18 years of age with thalassemia, other hemoglobinopathies, and hemolytic anemia were excluded from the study. The objectives of the study were (a) to understand the frequency of hypothyroidism in children aged between two and 18 years of age with beta-thalassemia major and (b) to gain knowledge of the relationship between thyroid function and serum ferritin levels. The study subjects were evaluated in detail and data were filled in a preformed structured proforma. A detailed history, general examination, and systemic examination were carried out. After the child was kept nil by mouth for 12 hours, an early morning sample of blood was collected. Three ml of blood was taken from the cubital vein under aseptic precautions, transferred to a plain bulb, and sent to the laboratory. It was assessed by the electrochemiluminescence technique for thyroid function test at the biochemistry department of the hospital.

Interpretation of thyroid profile

Subjects were classified into three groups according to their thyroid function test. Group I: Euthyroid: Normal T3, T4, and thyroid-stimulating hormone (TSH); Group II: Subclinical hypothyroidism: Normal T3, T4, and raised TSH. Group III: Overt hypothyroidism: decreased T3 or T4 and increased TSH. Normal thyroid function levels include: T3, 0.69-2.15 ng/ml; T4, 52-127 μg/ml; and TSH, 0.3-4.6 micro IU/ml. The data were filled in a Microsoft Excel (Microsoft, Redmond, WA) sheet and the association of these three groups was assessed. Data were analyzed with the help of IBM SPSS for Statistics, version 20.0 (IBM Corp., Armonk, NY.)

Ethics statement

Institutional clearance was obtained for the study from the institutional ethics committee (B.V.(D.U.) M C H/Sangli/IEC/431/21).

Statistical analysis

The chi-square test of independence was applied to study the correlation between serum ferritin and thyroid function tests.

## Results

Table 1 shows that in the present study 40 beta-thalassemia major patients were included. Of them, 18 (45%) patients were between six and 10 years, 15 (37.5%) patients were between 11 and 18 years and seven (17.5%) patients were between two and five years. In the study population, 24 (60%) patients were boys and 16 (40%) were girls.

**Table 1:** Age and gender-wise distribution

Age (Years)	Number (n = 40)	Percentage (%)	Boys	Percentage (%)	Girls	Percentage (%)
2-5	7	17.5	4	10	3	7.5
6-10	18	45	9	22	9	22
11-18	15	37.5	11	27	4	10

Table 2 shows thyroid function in these 40 study patients, in which 27 (67.5%) patients were euthyroid, eight (20%) patients had subclinical hypothyroidism, and five (12.5%) patients had overt hypothyroidism.

**Table 2:** Thyroid function in patients with B thalassemia

Thyroid function test	Number (n=40)	Percentage (%)
Euthyroid	27	67.5
Subclinical hypothyroidism	8	20
Overt hypothyroidism	5	12.5

Table 3 provides information about serum ferritin levels in the study patients. Serum ferritin level of >2000 ng/ml was seen in 26 patients, out of which 50% were euthyroid and 50% had hypothyroidism (19.23% had overt hypothyroidism and 30.77% had subclinical hypothyroidism).

**Table 3:** Comparison of serum ferritin with thyroid function

Serum ferritin (ng/ml)	Thyroid function test						Total	
	Euthyroid		Overt hypothyroidism		Subclinical hypothyroidism			
	n=27	%	n=5	%	n=8	%	n=40	%
<1000	5	100	0	0	0	0	5	12.5
1000-2000	9	100	0	0	0	0	9	22.5
>2000	13	50	5	19.23	8	30.77	26	65
Total	27	67.5	5	12.5	8	20	40	100

Table 4 shows that, in this study, there was a positive correlation between serum ferritin and hypothyroidism, as serum ferritin and TSH levels were increased, and the correlation was statistically significant (p<0.05).

**Table 4:** Correlation of serum ferritin with thyroid function test TSH: thyroid-stimulating hormone

Variable	Pearson’s correlation	P value
Serum ferritin and TSH	0.36	0.02
T4 and TSH	-0.41	0.009

## Discussion

In a repeatedly transfused thalassemic child, excess iron finally leads to multiple endocrine deficiencies causing hypothyroidism, diabetes mellitus, growth retardation, delayed or absent puberty, and osteoporosis [7]. Increased iron absorption as a response to chronic anemia adds to it. After an accumulation of 15-20 g excess iron in the body, iron-induced damage will manifest as liver cirrhosis, heart failure, and endocrine failure (pituitary, thyroid, parathyroid, and pancreas) [8]. Liver cirrhosis, heart failure, diabetes, and endocrine failure, also affecting the pituitary function, thyroid, parathyroid, pancreas, and gonadotropin will be evident clinically [8]. So, monitoring various endocrine dysfunctions should start ideally by five years or after three years of regular packed cell transfusion [9].

In our study subjects, 60% were boys and 40% girls. The male-to-female ratio was 1.5:1. In a study by Singhal and Goyal, the male-to-female ratio was 1.6:1 [10]. Considering the age of the studied subjects, 17.5.% were in the age group two to five years, 45% in six to 10 years age group, and 37.5% in the age group 11-18 years.

Our study showed hypothyroidism in 32.5% of the patients, of which 20% had subclinical hypothyroidism and 12.5% had overt hypothyroidism. In a study by Singhal and Goyal, 26.8% had subclinical hypothyroidism and 8% had overt hypothyroidism [10]. In a study conducted by Soliman et al., 35% prevalence of thyroidism was seen [11]. In this study, thyroid function was compared with serum ferritin levels and 30.77% of patients with subclinical hypothyroidism and 19.23% of patients with overt hypothyroidism had serum ferritin >2000 ng/ml. So this study showed a significant correlation between serum ferritin and hypothyroidism. As serum ferritin increased, TSH also increased and the correlation between hypothyroidism and these two parameters was statistically significant (p<0.05). Endocrine complications are commonly seen in adults [12]. But this study points out endocrine abnormality, especially hypothyroidism, in children with beta-thalassemia. With treatment of thyroid hormone replacement, the child resumes growth at a rate greater than normal [13]. After gonadotropin insufficiency, primary hypothyroidism is the most common endocrine abnormality. However, it is less common in patients who are on adequate chelation treatment [14]. 

Limitations of the study

A small sample size was the main limitation of our study. Also, this study was carried out at a single center. So multicenter studies with large sample sizes will be required to generalize the findings of this study.

## Conclusions

Hypothyroidism was found to be a complication in transfusion-dependent beta-thalassemia. Detection of hypothyroidism is important as effective hormone replacement therapy is available. Therefore, thyroid function should be assessed periodically, particularly in patients with high serum ferritin levels. Early recognition and treatment of hypothyroidism improve the quality of life of patients with beta-thalassemia.
